# *In Vitro α*-Amylase and *α*-Glucosidase Inhibitory and Antioxidant Activities of the Crude Extract and Solvent Fractions of *Hagenia abyssinica* Leaves

**DOI:** 10.1155/2021/6652777

**Published:** 2021-04-19

**Authors:** Zemene Demelash Kifle, Simachew Gidey Debeb, Yaschilal Muche Belayneh

**Affiliations:** ^1^Department of Pharmacology, School of Pharmacy, College of Medicine and Health Sciences, University of Gondar, Gondar, Ethiopia; ^2^Department of Clinical Pharmacy, School of Pharmacy, College of Medicine and Health Sciences, University of Gondar, Gondar, Ethiopia; ^3^Department of Pharmacy, College of Medicine and Health Sciences, Wollo University, Dessie, Ethiopia

## Abstract

**Background:**

The leaves of *Hagenia abyssinica* have been used in the management of diabetes mellitus in Ethiopian folk medicine. Thus, this study is aimed at investigating the *in vitro α*-amylase and *α*-glucosidase inhibitory and antioxidant activities of the crude extract and solvent fractions of *H. abyssinica* leaves.

**Methods:**

The *in vitro α*-amylase and *α*-glucosidase inhibitory and antioxidant activities of the plant extract were assessed using 3,5-dinitrosalicylic acid (DNSA), p-nitro-phenyl-a-D glucopyranoside (p-NPG), and 1,1-diphenyl-2-picrylhydrazyl (DPPH) assays, respectively. Each value of percent inhibition of *α*-amylase, *α*-glucosidase, and DPPH scavenging effect was presented as means ± SEM (*n* = 3).

**Results:**

The *α*-amylase inhibitory activity of the crude extract and solvent fractions was found to be concentration-dependent. The strongest activity was exhibited by the crude extract at the highest concentration with a percentage inhibition of 74.52% (IC_50_, 14.52 *μ*g/ml) followed by water fraction 68.24% (IC_50_, 16.31 *μ*g/ml), ethyl acetate fraction 61.57% (IC_50_, 18.73 *μ*g/ml), and chloroform fraction 56.87% (IC_50_, 21.57 *μ*g/ml) of *H. abyssinica* leaves. In the *α*-glucosidase inhibition assay, the maximum activity was exhibited by the *aqueous* fraction 62.54% (IC_50_, 11.67 *μ*g/ml) followed by ethyl acetate fraction 54.97% (IC_50_, 15.89 *μ*g/ml), crude extract 46.79% (IC_50_, >16.5 *μ*g/ml), and chloroform fraction 36.44% (IC_50_, >16.5 *μ*g/ml). In the antioxidant assay, the crude extract exhibited the highest antioxidant activity 86.36% (IC_50_, 10.25 *μ*g/ml) followed by water fraction 78.59% (IC_50_, 13.86 *μ*g/ml), ethyl acetate fraction 71.58% (IC_50_, 16.34 *μ*g/ml), and chloroform fraction 63.65% (IC_50_, 18.83 *μ*g/ml).

**Conclusion:**

This study has revealed that *H. abyssinica* leaves possess noticeable *in vitro α*-amylase and *α*-glucosidase inhibitory and antioxidant activities.

## 1. Background

Diabetes mellitus (DM) is a metabolic disorder of protein, fat, and carbohydrate metabolism attributed to low production of insulin or resistance to its action leading to hyperglycemia [1–3]. All over the world, one of the leading causes of morbidity as well as mortality is DM. Globally, the number of people with DM has increased from 108 million in 1980 to 422 million in 2014 [4]. In 2017, around 5 million deaths were reported due to DM in the 20–99 years age range [5]. The increasing prevalence of DM and adverse effects related to modern medications are also important points of apprehension [6].

Acarbose is a medication clinically used to inhibit *α*-glucosidase and *α*-amylase. Unfortunately, its long-term administration resulted in side effects including abdominal distention and diarrhea [7]. Alternative plant-derived products with better safety potential may also be used for the management of diabetes mellitus [7].

Oxidative stress is a condition arising due to excessive production of free radicals inside the body that leads to oxidation of biologically important molecules [8]. Chronic hyperglycemia increases the synthesis of nonmitochondrial and mitochondrial reactive oxygen species, leading to the stimulation of polyol pathway flux, hexosamine pathway flux, protein kinase C (PKC) isoforms, and advanced glycation end-products (AGE) associated with hyperglycemia-induced oxidative damage [9, 10]. Similarly, the amplified production of reactive oxygen species has harmful regulation of insulin signaling cascade leading to *β*-cell dysfunction, mitochondrial dysfunction, decreased insulin gene expression, impaired glucose tolerance, and insulin resistance [9, 11].

Natural plant-derived drugs are believed to be safe, effective, and economical [3]. Plant-derived products play a significant role in the development of new therapeutic agents and serve as sources of bioactive substances including antioxidants [12–14]. The antioxidant activity of plant extracts has valuable effects on the conservation of *β*-cell function in DM [15–17]. Currently, more than 1,000 herbal medicines have been pronounced as efficacious in the management of DM [18]. *Hagenia abyssinica* is the only species of the genus *Hagenia* that belongs to the Rosaceae family [19]. The species is found in Burundi, Congo, Ethiopia, Uganda, Kenya, Malawi, Sudan, Rwanda, and Tanzania [20]. Rosaceae is a huge family comprising more than 2,000 species and 100 genera [21]. Among the medicinal plants with confirmed antidiabetic effect used in the management of DM are *Elaeagnus umbellata* [22], *Eryngium caeruleum* [3], *Momordica charantia*, *Eugenia jambolana*, *Aloe vera*, *Ficus benghalensis*, *Ocimum sanctum syn.*, *Trigonella*, *Stevia rebaudiana*, *Coccinia indica*, *Allium sativum*, *Vernonia amygdalina*, *Falcaria vulgaris*, and *Foenum graecum* [17, 23–25].

Traditionally, the leaves of *H. abyssinica* have been used in the treatment of cough, wound, cancer, diarrhea, bone fracture, typhoid, hypertension, allergy, and livestock disease [26, 27]. Additionally, *H. abyssinica* has been used in the management of DM in Ethiopian folk medicine [28–30]. In the previous study, the leaf crude extract of *H. abyssinica* showed significant antidiabetic (streptozotocin-induced model) and antihyperlipidemic activities in mice [31]. However, there is no previous study on the *in vitro α*-amylase and *α*-glucosidase inhibitory and antioxidant activity of the leaves of the crude extract and solvent fractions of *H. abyssinica*. Therefore, this study was conducted to investigate the *in vitro α*-amylase and *α*-glucosidase inhibitory and antioxidant activity of the leaf crude extract and solvent fractions of *H. abyssinica*.

## 2. Methods

### 2.1. Plant Materials

The fresh leaves of *H. abyssinica* were collected from Tara Gedam, South Gondar zone, Amhara region, in December 2018. The botanical identification and authentication of *H. abyssinica* were done by a botanist, and the voucher specimen (0/2/Z-D-K/19) was deposited in the Biology Department, University of Gondar, Ethiopia.

### 2.2. Preparation of the Plant Extract and Solvent Fractionation

The preparation of the plant extract and solvent fractionations were done according to the method described by Kifle et al. [32]. The leaves of *H. abyssinica* were thoroughly washed with distilled water to remove dirt and dried at room temperature (25–27°C). The collected leaves of *H. abyssinica* were powdered through an electrical mill. Then, the milled leaves were macerated distinctly in 80% methanol for about three days and then filtered through Whatman filter paper. Likewise, fresh methanol was used to remacerate the marc, and then, the filtrate of each successive maceration was concentrated by using a rotary evaporator. Lastly, the semidried residue was frozen in the refrigerator and dried using a lyophilizer (Labfreez, China) to entirely confiscate the remaining solvent. Solvent fractionation of the leaf extract of *H. abyssinica* was carried out using water, ethyl acetate, and chloroform. Briefly, the leaf extract of *H. abyssinica* was dissolved in 400 ml of distilled water and this solution was transferred to a separating funnel. An equal volume of chloroform was added to it and was shaken vigorously. The mixture was separated into two layers, and then, the chloroform fraction was removed. The partition with chloroform was repeated two times. The chloroform layer was combined and subjected to evaporation using a hot air oven set at 40°C to get the dried chloroform fraction. Finally, 400 ml of ethyl acetate was added to the separating funnel containing the aqueous layer. The mixture was separated into two layers, and then, the ethyl acetate was separated and the procedure was repeated two times. The ethyl acetate fraction was pooled and concentrated using a hot air oven set at 40°C to obtain the dried ethyl acetate fraction. The remaining aqueous layer was concentrated using a hot air oven set at 40°C overnight and then concentrated in a lyophilizer to remove the water. After drying, the solvent fractions were stored in a desiccator until used for the experiment.

### 2.3. Determination of *α*-Amylase Inhibitory Activity

The *α*-amylase inhibition activity was done according to the method described by Kifle et al. [32]. The extract of *H. abyssinica* was dissolved in buffer ((NaCl (0.006 M), Na_2_HPO_4_/NaH_2_PO_4_ (0.02 M)) at pH 6.9 to make the concentrations (15.625 to 500 *μ*g/ml). 200 *μ*l of *α*-amylase solution (2 units/ml) was mixed with 200 *μ*l of the crude extract and solvent fractions and incubated for about 10 minutes at 30°C. Subsequently, 200 *μ*l of one % starch solution was added to each tube and incubated for about 3 minutes. Then, the reaction was terminated through 200 *μ*l DNSA reagent and was boiled for about 10 minutes using a water bath at 85°C. Finally, the mixture was cooled to ambient temperature and diluted with 5 ml of distilled water, and the absorbance was determined at 540 nm using a UV-visible spectrophotometer. The blank with 100% enzyme activity was prepared by substituting the extract with 200 *μ*l of the buffer. A blank with the plant extract at each concentration was used in the absence of the enzyme solution. A positive control sample was prepared using acarbose, and the reaction was done correspondingly to the reaction with extract as mentioned above.

The inhibition of alpha-amylase was articulated as a percent of inhibition and determined using the following equation: inhibition (%) = [(Ac − Acb) − (As − Asb)/(Ac − Acb)] × 100, where Ac is the absorbance of control, Acb is the absorbance of control blank, As is the absorbance of the sample, and Asb is the absorbance of sample blank. The IC_50_ values of the crude extract, solvent fractions, and acarbose were calculated from the dose-response curve through interpolation from the linear regression analysis.

### 2.4. *In Vitro α*-Glucosidase Inhibition Assay

The *in vitro α*-glucosidase inhibitory activity was done according to the method described by Tao et al. [33]. The *α*-glucosidase inhibitory effect of the crude extract and solvent fractions of *H. abyssinica* was evaluated by using p-nitro-phenyl-a-D glucopyranoside (p-NPG) substrate solution (using 0.1 M potassium phosphate buffer, pH 6.8). Similarly, 0.1 unit/ml of alpha-glucosidase was dissolved using potassium phosphate buffer. All the samples were dissolved in dimethyl sulfoxide, and 20 ml of each sample was mixed with the same volume of enzyme solution. Subsequently, a volume of 40 ml substrate solution was added for initiation of the reaction and incubated at 37°C for 40 minutes. Then, 80 ml of 0.2 M sodium carbonate in phosphate buffer, pH 6.8 was added to terminate the reaction. Finally, the amount of released p-nitrophenol (pNP) was measured at 405 nm. The *α*-glucosidase inhibitory activity was expressed as percent inhibition and determined as follows: %inhibition = [(average A 405 control − average A 405 extract)/average A 405 control] × 100. The IC_50_ values of the crude extract, solvent fractions, and acarbose were calculated from the dose-response curve through interpolation from the linear regression analysis.

### 2.5. Evaluation of *In Vitro* Antioxidant Activity Using the DPPH Assay

The free radical scavenging activity of the plant crude extract, solvent fractions, and ascorbic acid was done according to the method described by Kifle et al. [32]. Aliquots of 100 *μ*l of a methanolic solution comprising different concentrations of the plant extract and solvent fractions ranging from 15.625 to 500 *μ*g/ml were added to 3.9 ml of a 0.004% solution of 1,1-diphenyl-2-picrylhydrazyl. Finally, after 30 minutes, the absorbance at 517 nm was determined and the % inhibition of the extracts and the standard drug was calculated. The IC_50_ values of the extract and the standard drug represent the concentration of the sample required to scavenge 50% DPPH free radicals. The percentage (%) of DPPH free radical scavenging was calculated by the formula: (*A*_0_ − *A*_1_)/*A*_0_ × 100. *A*_0_ is the absorbance of the control, and *A*_1_ is the absorbance of the crude extract, solvent fractions, and standard drug.

### 2.6. Statistical Analysis

All the findings were expressed as mean ± standard error of the means (*n* = 3) for each value of percent inhibition of *α*-amylase, *α*-glucosidase, and DPPH scavenging activities. The IC_50_ values were calculated from the dose-response curve through interpolation from the linear regression analysis.

## 3. Results

### 3.1. The Percentage Yield of Plant Material Extraction

A total of 193.5 (14.6% *w*/*w*) grams of dried crude leaf extract of *H. abyssinica* was collected at the end of the extraction process. The yields of the fractions were 17.5% *w*/*w* (26.8 g), 29.8% *w*/*w* (45.6 g), and 47.8% *w*/*w* (73.1 g) for the chloroform, ethyl acetate, and aqueous solvent fraction, respectively (Table 1).

### 3.2. *In Vitro α*-Amylase Inhibition Activity of the Crude Extract and Solvent Fractions

**Figure 1:**
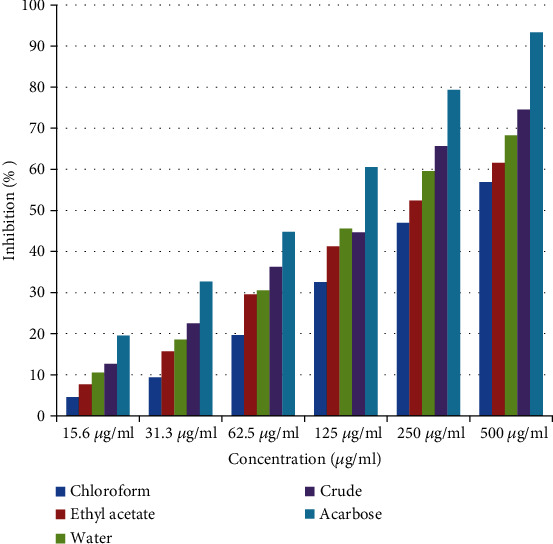
*α*-Amylase inhibitory activity of the leaf crude extract and solvent fractions of *Hagenia abyssinica.*

As shown in Figure 1, the crude extract and solvent fractions of *H. abyssinica* leaves exhibited a concentration-dependent reduction in percentage inhibition against *α*-amylase activity. In the *α*-amylase inhibition assay, the crude extract was observed to be the most active fraction. The crude extract exhibited a percentage inhibition of 12.64%, 22.52%, 36.25%, 44.65%, 65.67%, and 74.52% at concentrations of 15.6, 31.3, 62.5, 125.0, 250.0, and 500 *μ*g/ml, respectively, attaining IC_50_ of 14.52 *μ*g/ml. The water fraction displayed 12.64%, 22.51%, 36.25%, 44.65%, 65.67%, and 74.52% inhibitions at the same tested concentrations with an IC_50_ value of 16.31 *μ*g/ml, respectively. The ethyl acetate fraction exhibited 7.65%, 15.68%, 29.54%, 41.25%, 52.36%, and 61.57% inhibitions at the same tested concentrations with an IC_50_ value of 18.73 *μ*g/ml, respectively. Likewise, the chloroform fraction exhibited 4.58%, 9.36%, 19.65%, 32.58%, 46.95%, and 56.87% inhibitions at the same tested concentrations with an IC_50_ value of 21.57 *μ*g/ml, respectively. In comparison, the standard drug acarbose displayed 19.52%, 32.68%, 44.78%, 60.54%, 79.35%, and 93.34% inhibitions at concentrations of 15.6, 31.3, 62.5, 125.0, 250.0, and 500 *μ*g/ml, respectively, attaining IC_50_ of 9.82 *μ*g/ml (Table 2).

### 3.3. *In Vitro α*-Glucosidase Inhibition Activity

**Figure 2:**
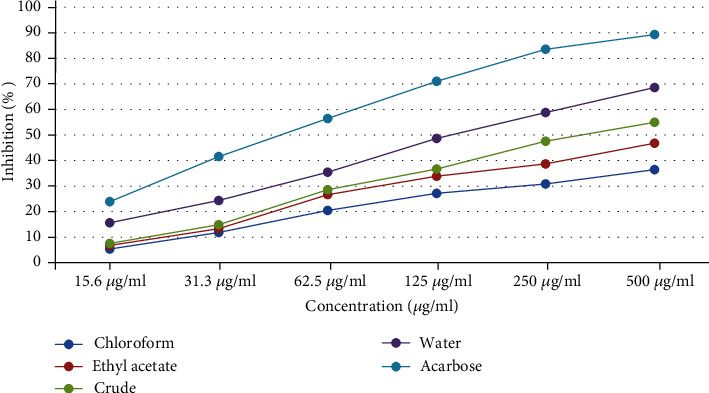
*α*-Glucosidase inhibitory activity of the crude extract and solvent fractions of *Hagenia abyssinica.*

The *in vitro α*-glucosidase inhibitory activity of the crude extract and solvent fractions of *H. abyssinica* was noticeable as shown in Table 3. The percentage inhibition of the crude extract and solvent fractions at concentrations of 15.625 to 500 *μ*g/ml was concentration-dependent (Figure 2). Accordingly, at the highest concentration (500 *μ*g/ml) of the crude extract and solvent fractions showed maximum percentage inhibition against *α*-glucosidase: 36.44% (IC_50_, >16.5 *μ*g/ml), 46.79% (IC_50_, >16.5 *μ*g/ml), 54.97 (IC_50_, 15.89 *μ*g/ml), 68.56% (IC_50_, 11.67 *μ*g/ml), and 89.32% (IC_50_, 6.45 *μ*g/ml), respectively, for chloroform fraction, ethyl acetate fraction, crude extract, aqueous fraction, and acarbose, while at the lowest concentration (15.6 *μ*g/ml) showed the lowest percentage inhibition, 5.34%, 6.74%, 15.64%, and 23.92%, respectively, for chloroform fraction, ethyl acetate fraction, crude extract, aqueous fraction, and acarbose. Moreover, the IC_50_ values of the crude extract and solvent fractions of *H. abyssinica* varied from 6.45 to >16.5 *μ*g/ml (Table 3).

### 3.4. Antioxidant Activity of the Crude Extract and Solvent Fractions

**Figure 3:**
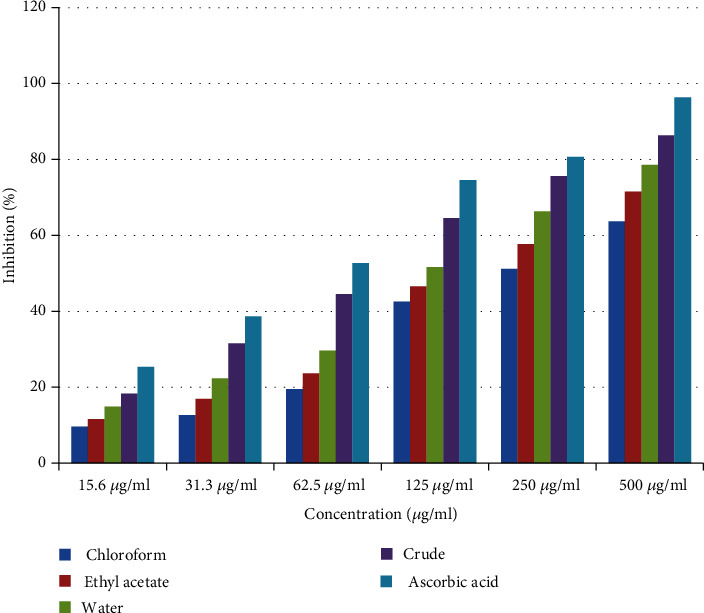
Free radical scavenging activity of the crude extract and solvent fractions of *Hagenia abyssinica.*

The antioxidant activity of the crude extract and solvent fractions was verified using 1,1-diphenyl-2-picrylhydrazyl free radical assay. The crude extract and solvent fractions displayed noticeable antioxidant activity as summarized in Table 4. There was a concentration-dependent rise in the percentage inhibition of free radicals at concentrations tested (15.6 to 500 *μ*g/ml) as shown in Figure 3. In DPPH free radical scavenging assay, the crude extract showed 18.36%, 31.56%, 44.58%, 64.57%, 75.61%, and 86.36% inhibitions of free radicals at concentrations of 15.6, 31.3, 62.5, 125.0, 250.0, and 500 *μ*g/ml, respectively, with IC_50_ of 10.25 *μ*g/ml. The water fraction displayed 14.92%, 22.36%, 29.64%, 51.64%, 66.35%, and 78.59% inhibitions at concentrations 15.6, 31.3, 62.5, 125.0, 250.0, and 500 *μ*g/ml, respectively, with IC_50_ of 13.86 *μ*g/ml. Similarly, the ethyl acetate fraction enzyme inhibitions were 11.65%, 16.94%, 23.67%, 46.58%, 57.64%, and 71.58%, respectively, at the same tested concentrations with IC_50_ of 16.34 *μ*g/ml. Furthermore, the chloroform fraction displayed 9.64%, 12.67%, 19.54%, 42.57%, 51.24%, and 63.65%, respectively, at the same tested concentrations with IC_50_ of 18.83 *μ*g/ml. In comparison, the standard drug ascorbic acid showed 25.36%, 38.67%, 52.67%, 74.58%, 80.67%, and 96.37% inhibition of free radicals at concentrations 15.6, 31.3, 62.5, 125.0, 250.0, and 500 *μ*g/ml, respectively, with IC_50_ of 7.39 *μ*g/ml (Table 4).

## 4. Discussion

Various phytoconstituents isolated from medicinal plants having *in vitro α*-glucosidase and *α*-amylase inhibitory and free radical scavenging activities have shown equivalent *α*-glucosidase and *α*-amylase inhibitory and free radical scavenging activities and sometimes more potent than known conventional agents [34–37]. This study was conducted to investigate the *in vitro α*-amylase and *α*-glucosidase inhibitory and antioxidant activity of the crude hydromethanolic extract and solvent fractions of *H. abyssinica* leaves.

The free radicals are frequently generated and cause extensive damage to biomolecules and tissue leading to numerous disease conditions, predominantly extensive lysis and degenerative diseases in the living systems [38]. Different medications have a protective effect against oxidative damage; nevertheless, they have several side effects. The use of plant-based antioxidants from traditional medicines plays a significant role in reducing the adverse side effects of existing drugs [39–41]. Currently, several antioxidants have been synthesized from various medicinal plant materials [42].

Inhibition of intestinal pancreatic *α*-glucosidase and *α*-amylase activities results in delayed carbohydrate digestion of absorbable monosaccharides leading to a drop in postprandial hyperglycemia [43]. In the present study, the crude extract and solvent fractions of *H. abyssinica* showed *α*-amylase inhibitory activity. The search for a new *α*-amylase inhibitor from medicinal plants is a striking method for the management of postprandial hyperglycemia. Secondary metabolites such as tannins, phenolic acids, and flavonoids are the main phytoconstituents that possess *α*-amylase inhibitory activity [44]. In the previous finding, the phytochemical analysis showed that crude extract and solvent fractions are rich in polyphenolic compounds with *α*-amylase inhibitory activity [45].

The crude extract and solvent fractions of *H. abyssinica* leaves exhibited noticeable *α*-amylase inhibitory activities: 56.87%, 61.57%, 68.24%, 74.52%, and 93.34%, respectively, for chloroform solvent fraction, ethyl acetate solvent fraction, water solvent fraction, crude extract, and acarbose at a concentration of 500 *μ*g/ml. In contrast, fairly weak *α*-amylase inhibitory activity (56.87%) was produced by the chloroform fraction, and the highest *α*-amylase inhibitory activity was recorded by the crude extract. According to the current findings, it was suggested that the *α*-amylase inhibitory activities of the extracts increased with increasing polarity of the solvent fractions (chloroform, ethyl acetate, and water solvent fractions). The current findings showed that the crude extract had an IC_50_ value of 14.52 ± 0.94 *μ*g/ml. This concentration was lower than for the solvent fractions (Table 2). Furthermore, it can be proposed that the crude extract possessed higher concentrations of phytoconstituents for *α*-amylase inhibition than the solvent fractions.

In the *in vitro α*-glucosidase inhibitory activity, the highest concentration (500 *μ*g/ml) of the crude extract and solvent fractions showed a maximum percentage of inhibition against *α*-glucosidase: 36.44%, 46.79%, 54.97%, 68.56%, and 89.32%, respectively, for chloroform fraction, crude extract, ethyl acetate fraction, *aqueous* fraction, and acarbose. This finding is in line with previous similar studies [46, 47], comparing the current findings with previous results in which the *α*-glucosidase IC_50_ value of banana flower extract (umbelliferone) was 7.79 ± 0.11 g/ml [48]. In the present study, the highest *α*-glucosidase inhibitory activity was shown by the crude extract and *aqueous* fraction (IC_50_: 15.89 *μ*g/ml and 11.67 *μ*g/ml, respectively).

Medicinal plants are usually rich in various phenolic compounds such as flavonoids, tannins, lignans, phenolic acids, coumarins, lignins, and stilbenes [49]. Inhibition of *α*-glucosidase is one of the therapeutic approaches for managing DM to reduce postprandial hyperglycemia (delaying the absorption of glucose). Alpha-glucosidase inhibitors have been used in the management of DM and denoted at the large ratio of the antidiabetic drug market [50]. The crude extract and solvent fractions are rich in phenolic compounds as reported in the literature, which may contribute to its *in vitro* antidiabetic effect. Furthermore, the *aqueous* fraction exhibited the highest *α*-glucosidase inhibitory activity (IC_50_: 11.67, 68.56%).

The activity of antioxidants on DPPH is due to their hydrogen-donating ability [51]. Free radicals play a significant role in biological damages, and DPPH has been used to assess the free radical scavenging activity of medicinal plants with antioxidant activities [52]. DPPH is a free radical with a purple color and changed into a stable compound with a yellow color when reacting with antioxidants [53]. The inhibitory activity of the crude extract and the solvent fractions against DPPH free radical was determined by the reduction in its absorbance, induced by antioxidants. At all concentrations tested, the crude extract of *Hagenia abyssinica* leaves showed a higher DPPH scavenging activity (18.36% at 15.6 *μ*g/ml and 86.36 at 500 *μ*g/ml) compared to the solvent fractions. This activity was lower in chloroform solvent fraction (9.64% at the concentration of 15.6 *μ*g/ml and 63.65% at the concentration of 500 *μ*g/ml) compared to the crude extract, ethyl acetate, and *aqueous* fractions. Ascorbic acid exhibited a percentage inhibition of 25.36% at a concentration of 15.625 *μ*g/ml and 96.37% at a concentration of 500 *μ*g/ml. This finding suggests that the leaf crude extract of *H. abyssinica* contains more phenolic compounds that may contribute to its antioxidant properties than the solvent fractions.

In the previous study, the preliminary phytochemical analysis revealed that the crude extract and solvent fractions of *H. abyssinica* contain various secondary metabolites such as alkaloids, flavonoids, triterpenoids, tannins, steroids, glycosides, phenols, saponins, and anthraquinones [45]. Phytochemicals isolated from different plant species have been reported to have potent antidiabetic activity [35, 36]. Secondary metabolites such as flavonoids, tannins, lignans, glycosides, phenolic acids, coumarins, lignins, and stilbenes were some of the reported compounds that were isolated from medicinal plants with a potential to inhibit *α*-amylase, *α*-glucosidase, and scavenge free radicals [34–37]. Thus, the significant *in vitro α*-amylase and *α*-glucosidase inhibitory and antioxidant activities of the crude extract and solvent fractions of *H. abyssinica* could be due to the presence of the aforementioned phytoconstituents in the leaves of the plant.

## 5. Conclusions

The crude extract and solvent fractions of *H. abyssinica* leaves have shown *in vitro* antioxidant, *α*-glucosidase, and *α*-amylase inhibitory activities. The phytoconstituents of the plant extract might contribute to the *in vitro* enzyme inhibitory and free radical scavenging activities. Thus, this study confirms that the leaves of *H. abyssinica* can mitigate postprandial hyperglycemia and ameliorate oxidative stress and therefore assist in combating diabetic complications. Further studies are recommended to substantiate the use of the plant as an antidiabetic agent.

## Figures and Tables

**Table 1:** Yields of 80% methanol crude extract and solvent fractions of *H. abyssinica.*

Extract	Actual yield (g)	Percentage yield
Methanol 80% crude extract	193.5	14.6%
Ethyl acetate fraction (EAF)	45.6	29.8%
Chloroform fraction (CHF)	26.8	17.5%
Aqueous fraction (AQF)	73.1	47.8%

**Table 2:** *α*-Amylase inhibitory activities of the crude extract and solvent fractions of *H. abyssinica.*

Concentration	Percentage (%) inhibition				
	Chloroform fraction	Ethyl acetate fraction	Water fraction	Crude extract	Acarbose
15.6 *μ*g/ml	4.58 ± 0.68	7.65 ± 0.88	10.54 ± 0.99	12.64 ± 0.85	19.52 ± 0.76
31.3 *μ*g/ml	9.36 ± 0.59	15.68 ± 0.67	18.54 ± 0.67	22.51 ± 1.64	32.68 ± 0.66
62.5 *μ*g/ml	19.65 ± 1.57	29.54 ± 0.49	30.54 ± 0.57	36.25 ± 0.58	44.78 ± 1.18
125.0 *μ*g/ml	32.58 ± 0.96	41.25 ± 1.34	45.58 ± 1.28	44.65 ± 0.67	60.54 ± 0.67
250.0 *μ*g/ml	46.95 ± 0.72	52.36 ± 0.75	59.54 ± 0.87	65.67 ± 0.93	79.35 ± 0.91
500.0 *μ*g/ml	56.87 ± 0.49	61.57 ± 0.49	68.24 ± 0.67	74.52 ± 0.80	93.34 ± 0.58
IC_50_ *μ*g/ml	21.57 ± 1.03	18.73 ± 0.87	16.31 ± 0.54	14.52 ± 0.94	9.82 ± 0.77

Note: each value of percent inhibition of *α*-amylase is presented as means ± SEM (*n* = 3). Abbreviations: SEM: standard error of mean; IC_50_: half-maximal inhibitory concentration.

**Table 3:** *α*-Glucosidase inhibitory activities of the crude extract and solvent fractions of *H. abyssinica*.

Concentration	Percentage (%) inhibition				
	Chloroform fraction	Ethyl acetate fraction	Crude extract	Water fraction	Acarbose
15.6 *μ*g/ml	5.34 ± 1.34	6.74 ± 0.86	7.56 ± 0.56	15.64 ± 1.13	23.92 ± 0.96
31.3 *μ*g/ml	11.83 ± 0.45	13.32 ± 1.25	14.87 ± 1.40	24.34 ± 0.55	41.54 ± 1.06
62.5 *μ*g/ml	20.46 ± 0.91	26.66 ± 0.94	28.57 ± 0.12	35.45 ± 0.83	56.44 ± 0.83
125.0 *μ*g/ml	27.14 ± 0.66	33.86 ± 1.11	36.66 ± 0.34	48.65 ± 0.91	71.01 ± 0.66
250.0 *μ*g/ml	30.84 ± 1.21	38.67 ± 0.54	47.56 ± 0.76	58.78 ± 1.44	83.56 ± 1.33
500.0 *μ*g/ml	36.44 ± 0.32	46.79 ± 0.83	54.97 ± 1.22	68.56 ± 0.03	89.32 ± 0.44
IC_50_ *μ*g/ml	16.5 ± 1.23	>16.5 ± 0.45	15.89 ± 0.65	11.67 ± 0.97	6.45 ± 1.04

Note: each value of percent inhibition of *α*-glucosidase is presented as means ± SEM (*n* = 3). Abbreviations: SEM: standard error of mean; IC_50_: half-maximal inhibitory concentration.

**Table 4:** Antioxidant activities of the crude extract and solvent fractions.

Concentration	Percentage (%) inhibition				
	Chloroform fraction	Ethyl acetate fraction	Water fraction	Crude extract	Ascorbic acid
15.6 *μ*g/ml	9.64 ± 1.23	11.65 ± 0.87	14.92 ± 0.37	18.36 ± 1.41	25.36 ± 0.67
31.3 *μ*g/ml	12.67 ± 0.96	16.94 ± 1.35	22.36 ± 0.86	31.56 ± 0.67	38.67 ± 0.59
62.5 *μ*g/ml	19.54 ± 0.58	23.67 ± 0.67	29.64 ± 1.34	44.58 ± 0.55	52.67 ± 0.75
125.0 *μ*g/ml	42.57 ± 1.30	46.58 ± 0.86	51.64 ± 0.94	64.57 ± 0.94	74.58 ± 1.36
250.0 *μ*g/ml	51.24 ± 0.67	57.64 ± 0.77	66.35 ± 0.58	75.61 ± 0.83	80.67 ± 0.93
500.0 *μ*g/ml	63.65 ± 0.54	71.58 ± 1.09	78.59 ± 0.93	86.36 ± 0.72	96.37 ± 0.87
IC_50_ *μ*g/ml	18.83 ± 0.96	16.34 ± 0.74	13.86 ± 0.88	10.25 ± 0.51	7.39 ± 0.49

Note: each value of percent inhibition of DPPH is presented as means ± SEM (*n* = 3). Abbreviations: SEM: standard error of mean; DPPH: 2,2-diphenyl-1-picrylhydrazine; IC_50_: half-maximal inhibitory concentration.

## Data Availability

The data sets used and/or analyzed during the current study are available from the corresponding author upon reasonable request.

## Abbreviations

AGE: Advanced glycation end-products (AGE)
DNSA: 3,5-Dinitrosalicylic acid
DM: Diabetes mellitus
DPPH: 1,1-Diphenyl-2-picrylhydrazyl
IC_50_: Half-maximal inhibitory concentration
PKC: Protein kinase C
p-NPG: p-Nitro-phenyl-a-D glucopyranoside
WHO: World Health Organization.
